# Supplementary material for: Performance of Chlorella Vulgaris Exposed to Heavy Metal Mixtures: Linking Measured Endpoints and Mechanisms
Source: Int J Environ Res Public Health. 2021 Jan 25;18(3):1037. doi: 10.3390/ijerph18031037 (PMC7908404; doi:10.3390/ijerph18031037)
Supplement: Supplementary file 1 [file ijerph-18-01037-s001.pdf]

Supplementary materials

Table S1. Metal speciation at the different treatment concentrations in single metal exposure expressed as % of total metal concentration for growth inhibition tests.

Species name	As 1.75 ppb	As 3.5 ppb	As 17.5 ppb	As 35 ppb
$HAsO_4^{-2}$	46.189	46.189	46.19	46.192
$H_2AsO_4^-$	53.808	53.808	53.807	53.806
**Species name**	**Pb 1 ppb**	**Pb 2 ppb**	**Pb 10 ppb**	**Pb 20 ppb**
Pb^{+2}	0.009	0.009	0.01	0.013
$PbEDTA^{-2}$	99.991	99.991	99.99	99.987
**Species name**	**Ni 1.35 ppb**	**Ni 2.7 ppb**	**Ni 13.5 ppb**	**Ni 64.8 ppb**
$NiEDTA^{-2}$	99.959	99.959	99.959	99.958
$NiHEDTA^-$	0.041	0.041	0.041	0.042
**Species name**	**Zn 10.5 ppb**	**Zn 21 ppb**	**Zn 105 ppb**	**Zn 210 ppb**
Zn^{+2}	0.019	0.021	0.047	0.17
$ZnHPO_4$ (aq)	0.01	0.011	0.025	0.092
$ZnEDTA^{-2}$	99.937	99.934	99.893	99.698
$ZnHEDTA^-$	0.032	0.032	0.032	0.032
**Species name**	**Cu 2.5 ppb**	**Cu 5 ppb**	**Cu 25 ppb**	**Cu 50 ppb**
$CuEDTA^{-2}$	99.958	99.958	99.958	99.956
$CuHEDTA^-$	0.04	0.04	0.04	0.04

For As the two species present were at nearly equal concentrations for all treatments. The As 3.5 ppb, Pb 2 ppb, Ni 2.7 ppb, Zn 21 ppb and Cu 5 ppb were the concentrations used in the heavy metal mixture of the mixture experiment in growth inhibition test.

Table S2. Metal speciation at the different treatment concentrations in single metal exposure expressed as % of total metal concentration for photosynthesis inhibition tests.

**Species name**	**Zn 0 ppb**	**Zn 6 ppb**	**Zn 65.4 ppb**	**Zn 653.8 ppb**	**Zn 3.3 ppm**	**Zn 6.5 ppm**	**Zn 13.1 ppm**	**Zn 26.2 ppm**	**Zn 32.7 ppm**	**Zn 65.4 ppm**	**Zn 131 ppm**	**Zn 327 ppm**	**Zn 654 ppm**
Zn^{+2}	0.02	0.02	0.03	1.56	47.85	55.52	59.77	62.71	63.60	66.74	71.16	79.29	85.45
Zn_2OH^{+3}													0.01
$ZnOH^+$					0.23	0.27	0.28	0.30	0.30	0.31	0.33	0.34	0.34
$Zn(OH)_2$ (aq)					0.02	0.02	0.02	0.02	0.02	0.02	0.02	0.02	0.02
$ZnCl^+$						0.10	0.11	0.14	0.15	0.22	0.35	0.73	1.29
$ZnSO_4$ (aq)				0.05	1.51	1.72	1.83	1.88	1.88	1.86	1.77	1.46	1.09
$ZnNO_3^+$					0.24	0.28	0.30	0.31	0.31	0.32	0.33	0.34	0.33
$ZnHPO_4$ (aq)			0.01	0.84	25.79	29.21	30.72	30.79	30.52	28.69	24.98	17.32	11.19
$ZnEDTA^{-2}$	99.94	99.94	99.93	97.51	24.36	12.88	6.95	3.85	3.20	1.83	1.05	0.49	0.27
$ZnHEDTA^-$	0.03	0.03	0.03	0.03									
**Species name**	**Cu 0 ppb**	**Cu 6 ppb**	**Cu 63 ppb**	**Cu 635 ppb**	**Cu 3.1 ppm**	**Cu 6.35 ppm**	**Cu 12.7 ppm**	**Cu 25.4 ppm**	**Cu 31.7 ppm**	**Cu 63.5 ppm**	**Cu 127 ppm**	**Cu 317 ppm**	**Cu 635 ppm**
Cu^{+2}					12.25	16.39	19.32	21.56	22.19	23.53	22.28	16.85	12.58
$CuOH^+$					1.85	2.47	2.91	3.24	3.33	3.51	3.26	2.34	1.63
Cu_2OH^{+3}										0.02	0.03	0.04	0.05
$Cu(OH)_2$ (aq)					0.02	0.03	0.03	0.03	0.04	0.04	0.03	0.02	0.02
$Cu_2(OH)_2^{+2}$					0.13	0.47	1.30	3.23	4.27	9.50	16.62	22.16	22.57
$Cu_3(OH)_4^{+2}$						0.02	0.10	0.58	0.98	4.56	14.73	34.65	48.11
$CuCl^+$					0.01	0.02	0.02	0.02	0.02	0.02	0.02	0.02	0.01
$CuSO_4$ (aq)					0.43	0.60	0.79	1.04	1.16	1.66	2.29	3.07	3.53
$CuNO_3^+$					0.08	0.10	0.12	0.14	0.14	0.15	0.13	0.09	0.06
$CuHPO_4$ (aq)				0.03	41.89	54.85	61.81	63.06	62.14	54.12	39.14	20.16	11.14
$CuEDTA^{-2}$	99.96	99.96	99.96	99.92	43.33	25.04	13.59	7.10	5.73	2.92	1.47	0.59	0.30
$CuHEDTA^-$	0.04	0.04	0.04	0.04	0.02	0.01							
**Species name**	**Ni 0 ppb**	**Ni 6 ppb**	**Ni 58 ppb**	**Ni 587 ppb**	**Ni 2.94 ppm**	**Ni 5.87 ppm**	**Ni 11.7 ppm**	**Ni 23.5 ppm**	**Ni 29.4 ppm**	**Ni 58.7 ppm**	**Ni 117 ppm**	**Ni 294 ppm**	**Ni 587 ppm**
Ni^{+2}				0.16	44.50	59.50	68.75	74.22	75.50	78.82	81.97	86.85	90.72
$NiOH^+$					0.03	0.04	0.04	0.04	0.05	0.05	0.05	0.05	0.04
$NiCl^+$						0.01	0.02	0.02	0.02	0.03	0.05	0.10	0.17
$NiSO_4$ (aq)					1.26	1.68	1.91	2.00	2.00	1.95	1.78	1.38	0.99
$NiNO_3^+$					0.22	0.30	0.34	0.36	0.37	0.38	0.38	0.36	0.34
$NiH_2PO_4^+$					0.27	0.36	0.40	0.42	0.42	0.41	0.37	0.28	0.20

$NiHPO_4$ (aq)				0.04	10.37	13.74	15.60	16.27	16.28	15.66	14.04	10.44	7.26
$NiEDTA^{-2}$		99.96	99.96	99.76	43.33	24.38	12.93	6.66	5.36	2.71	1.36	0.55	0.27
$NiHEDTA^{-}$		0.04	0.04	0.04	0.02								
**Species name**	**Pb 51.8 ppb**	**Pb 2590 ppb**	**Pb 5.18 ppm**	**Pb 10.36 ppm**	**Pb 20.72 ppm**	**Pb 41.44 ppm**							
Pb^{+2}	0.009	3.465	23.767	38.677	48.747	55.188							
$PbOH^{+}$		0.416	2.851	4.636	5.835	6.588							
$Pb(OH)_2$ (aq)					0.011	0.012							
Pb_2OH^{+3}					0.013	0.034							
$PbCl^{+}$		0.068	0.479	0.815	1.113	1.456							
$PbSO_4$ (aq)		0.242	1.659	2.687	3.353	3.721							
$PbNO_3^{+}$		0.102	0.697	1.134	1.426	1.607							
$PbH_2PO_4^{+}$		0.078	0.537	0.871	1.088	1.212							
$PbHPO_4$ (aq)		1.134	7.763	12.578	15.714	17.476							
$PbEDTA^{-2}$	99.991	94.486	62.229	38.576	22.684	12.686							
**Species name**	**As 0.1 ppb**	**As 10000 ppb**											
$HAsO_4^{-2}$	46.19	46.30											
$H_2AsO_4^{-}$	53.81	53.70											

For As the two species present were at nearly equal concentrations for all treatments.

Table S3. Metal speciation at the different treatment concentrations in mixture metal exposure expressed as % of total metal concentration for photosynthesis inhibition tests.

**Species name**	**Cu 19.9ppb**	**Cu 178.7ppb**	**Cu 3.2ppm**	**Cu 8.0 ppm**	**Cu 15.9 ppm**	**Cu 31.8 ppm**	**Cu 79.4ppm**	**Cu 158.8ppm**
Cu^{+2}			17.27	20.94	23.48	26.51	28.85	26.24
$CuOH^{+}$			2.60	3.12	3.45	3.79	3.85	3.20
Cu_2OH^{+3}							0.03	0.05
$Cu(OH)_2^{-}$(aq)			0.03	0.03	0.04	0.04	0.04	0.03
$Cu_2(OH)_2^{+2}$			0.26	0.94	2.32	5.71	15.38	22.58

$Cu_3(OH)_4^{+2}$				0.05	0.27	1.46	9.75	23.10
$CuCl^{+}$			0.03	0.04	0.07	0.13	0.27	0.42
$CuSO_4$ (aq)			0.55	0.65	0.69	0.70	0.58	0.38
$CuNO_3^{+}$			0.11	0.13	0.14	0.16	0.15	0.12
$CuHPO_4$ (aq)			57.39	64.76	64.66	58.95	40.06	23.40
$CuEDTA^{-2}$	99.96	99.95	21.75	9.34	4.88	2.55	1.04	0.48
$CuHEDTA^{-}$	0.04	0.04						
**Species name**	**Ni 0 ppb**	**Ni 146.8 ppb**	**Ni 2.9 ppm**	**Ni 7.3 ppm**	**Ni 14.7 ppm**	**Ni 29.4 ppm**	**Ni 73.4ppm**	**Ni 146.8ppm**
Ni^{+2}			55.89	69.76	76.38	81.73	88.07	91.78
$NiOH^{+}$			0.03	0.04	0.05	0.05	0.05	0.05
$NiCl^{+}$			0.02	0.03	0.04	0.07	0.16	0.27
$NiSO_4$ (aq)			1.56	1.88	1.95	1.88	1.55	1.17
$NiNO_3^{+}$			0.28	0.34	0.37	0.38	0.37	0.34
$NiH_2PO_4^{+}$			0.33	0.38	0.37	0.32	0.22	0.15
$NiHPO_4$ (aq)			12.55	14.59	14.22	12.29	8.27	5.53
$NiEDTA^{-2}$		99.96	29.34	12.97	6.62	3.28	1.32	0.70
$NiHEDTA^{-}$		0.04	0.01					
**Species name**	**Zn 53.9ppb**	**Zn 217.3ppb**	**Zn 3.3ppm**	**Zn 8.2ppm**	**Zn 16.4 ppm**	**Zn 32.7ppm**	**Zn 81.8ppm**	**Zn 163.5ppm**
Zn^{+2}	0.45	0.45	64.04	65.65	68.05	72.13	79.55	84.50
Zn_2OH^{+3}								
$ZnOH^{+}$			0.30	0.31	0.32	0.33	0.34	0.33
$Zn(OH)_2$ (aq)			0.02	0.02	0.02	0.02	0.02	0.02
$ZnCl^{+}$			0.14	0.19	0.29	0.50	1.09	1.96
$ZnCl_3^{-}$								
$ZnCl_2$ (aq)								0.02
$ZnSO_4$ (aq)	0.01	0.01	1.96	1.94	1.91	1.82	1.54	1.18
$ZnNO_3^{+}$			0.32	0.32	0.33	0.33	0.34	0.32
$ZnHPO_4$ (aq)	0.24	0.24	32.96	31.46	29.03	24.84	17.11	11.67
$ZnEDTA^{-2}$	99.26	99.26	0.26	0.10	0.05	0.02		
$ZnHEDTA^{-}$	0.03	0.03						

Table S4. Parameters and fit goodness coefficients of growth inhibition concentration-response curves for single heavy metal exposed samples.

C. vulgaris test growth inhibition (single metals)	**Heavy metal**	**Chemical compound used in the test**	**Heavy metal chemical species**	a	b	c	d	e	f	SSE	R2	Adj R2	RMSE	DFE
Growth 100% availability	As	$HAsNa_2O_4$	$HAsO_4^{-2}$ $H2AsO^{-4}$	181.996	-180.033	49.873	77.748	0.272	0.070	20933.8	0.686	0.573	38.669	14
	Cu	$CuSO_4$	$CuEDTA^{-2}$ $CuHEDTA^{-}$	86.580	-86.580	0.150	1.330	0.152	1.311	15438.8	-0.118	-0.517	33.208	14
	Ni	$NiCl_2$	$NiHEDTA^{-}$ $NiEDTA^{-2}$	1262.86	-1260.90	195.711	1425.12	0.091	-2.461	14281.6	0.514	0.340	31.939	14
	Pb	$PbCl_2$	$PbEDTA^{-2}$ Pb^{+2}	807.000	-807.000	2.633	35.712	0.100	-1.990	9552.1	0.626	0.492	26.121	14
	Zn	$ZnCl_2$	Zn^{+2} $ZnHPO_4$ (aq) $ZnEDTA^{-2}$ $ZnHEDTA^{-}$	67.052	-65.552	2.270	5.996	-62273.8	97742.2	6317.0	0.470	0.280	21.242	14
Growth Free Ions	As	$HAsNa_2O_4$	$HAsO_4^{-2}$ $H2AsO_4^{-}$	181.996	-180.033	49.873	77.748	0.272	0.070	20933.8	0.686	0.573	38.669	14
	Pb	$PbCl_2$	Pb^{+2}	9154.173	-9152.173	19.400	5.100	0.161	-5.127	12224.343	0.521	0.350	29.549	14
	Zn	$ZnCl_2$	Zn^{+2}	88.080	-88.080	448048.993	205588.400	0.4	-0.1	6898.1	0.421	0.214	22.197	14

Hormetic model (x) = ((a- b)./(1+(exp((c* x)-d)))))./(1+(exp((e*x)- f))). Fit parameters in Matlab: MaxFunEvals = 6000; MaxIter = 40000; Robust = 'Bisquare'.

Table S5. Parameters and fit goodness coefficients of photosynthesis inhibition concentration-response curves for single heavy metal exposed samples

C. vulgaris test photo-synthesis inhibition (single metals)	**Heavy metal**	**Chemical compound used in the test**	**Heavy metal chemical species**	a	b	c	d	e	f	SSE	R2	Adj R2	RMSE	DFE
Acute PAR 83	Cu	$CuSO_4$	Cu^{+2}, $CuSO_4$, $Cu(OH)_2$, $CuOH^+$, $CuHPO_4$	0.559	-0.049	2.113	10.747	-0.023	-0.163	0.003	0.930	0.885	0.020	8
24h PAR 283				2.050	-1.540	17.276	83.190	-0.021	-3.029	0.005	0.908	0.850	0.026	8
24h PAR 83				0.840	-0.330	18.122	88.019	-0.009	-0.757	0.004	0.984	0.974	0.023	8
Acute PAR 83			Cu^{+2}, $CuSO_4$, $Cu(OH)_2$, $CuOH^+$	0.649	-0.139	1.453	6.830	-0.038	-0.582	0.004	0.917	0.865	0.022	8
24h PAR 283				2.194	-1.684	12.706	55.770	-0.022	-3.101	0.004	0.932	0.890	0.022	8
24h PAR 83				2.185	-1.675	13.231	58.852	-0.007	-2.230	0.005	0.982	0.971	0.024	8
Acute PAR 83	Ni	$NiCl_2$	Ni^{+2}	2.181	-1.871	-16.390	41.218	-0.039	-2.476	0.010	0.460	0.122	0.035	8
24h PAR 283				0.468	-0.158	10.886	56.360	-0.008	-1.092	0.003	0.927	0.881	0.018	8
24h PAR 83				0.364	-0.054	2.721	16.040	0.129	9.139	0.002	0.935	0.895	0.017	8
Acute PAR 83	Zn	$ZnCl_2$	Zn^{+2}	2.174	-1.864	-2.007	79.429	-0.026	-2.368	0.010	0.350	-0.056	0.036	8
24h PAR 283				0.763	-0.453	2.623	13.115	-0.027	-1.762	0.010	0.750	0.593	0.035	8
24h PAR 83				0.867	-0.557	1.786	10.562	-0.015	-0.921	0.007	0.806	0.685	0.029	8

Hormetic model (x) = ((a- b)./(1+(exp((c* x)-d)))).(1+(exp((e*x)- f))). Fit parameters in Matlab: MaxFunEvals = 6000; MaxIter = 40000; Robust = 'Bisquare'.

Table S6. Parameters and fit goodness coefficients of photosynthesis and growth inhibition concentration-response curves for mixture heavy metals exposed samples and AC and IA models.

C. vulgaris (mixture metals)	**Heavy metals mix**	**Chemical compound used in the test**	**Heavy metal chemical species**	a	b	c	d	e	f	SSE	R2	Adj R2	RMSE	DFE
Acute PAR 83	Cu + Ni + Zn + As	$CuSO_4$ + $NiCl_2$ + $ZnCl_2$ + $HAsNa_2O_4$	All species except complexed with EDTA and PO_4	1.51	-1.15	2.93	17.34	0.02	-2.60	0.002	0.85	0.78	0.01	10
Acute PAR 283				0.25	0.06	2.20	12.87	0.08	0.17	0.003	0.66	0.49	0.02	10
24h PAR 83				0.30	0.01	-11.43	78.61	1.66	7.62	0.008	0.94	0.91	0.03	10
24h PAR 283				0.93	-0.62	1.11	4.71	-0.04	-2.46	0.002	0.91	0.87	0.01	10
CA model 24h PAR 83	Cu + Ni + Zn	$CuSO_4$ + $NiCl_2$ + $ZnCl_2$	All species except complexed with EDTA and PO_4	1.10	-0.10	-0.02	-1.14	3.18	17.47	0.002	0.97	0.93	0.02	3
CA model 24h PAR 283				0.92	0.08	0.00	-1.78	6.28	31.14	0.000	0.99	0.97	0.01	3
IA model 24h 83 PAR	Cu + Ni + Zn	$CuSO_4$ + $NiCl_2$ + $ZnCl_2$	All species except complexed with EDTA and PO_4	3.74	-2.94	3.61	20.45	-0.01	-3.08	0.000	1.00	0.99	0.01	3
IA model 24h PAR 283				2.20	-0.80	-0.02	-3.19	9.58	49.85	0.000	0.99	0.98	0.01	4
CA model growth with nominal conc	Cu + Ni + Zn + As + Pb	$CuSO_4$ + $NiCl_2$ + $ZnCl_2$ + $HAsNa_2O_4$ + $PbCl_2$	100% bioavailabil-ity	492.80	270.50	0.50	1.17	25.67	62.44	69.86	1.00	1.00	4.18	4
CA model growth with bioavailabable conc	Zn + As + Pb	$ZnCl_2$ + $HAsNa_2O_4$ + $PbCl_2$	$HAsO_4^{-2}$ $H_2AsO_4^-$ Pb^{+2} Zn^{+2}	-164.300	-459.300	0.271	0.049	23.180	36.320	33.008	0.999	0.998	2.873	4
IA model growth with bioavailabable conc	Zn + As + Pb	$ZnCl_2$ + $HAsNa_2O_4$ + $PbCl_2$	$HAsO_4^{-2}$ $H_2AsO_4^-$ Pb^{+2} Zn^{+2}	476.123	228.876	28.570	44.848	0.707	-0.642	84.499	0.999	0.997	4.596	4

Hormetic model (x) = ((a- b)./(1+(exp((c* x)-d)))./(1+(exp((e*x)- f))). Fit parameters in Matlab: MaxFunEvals = 6000; MaxIter = 40000; Robust = 'Bisquare'.

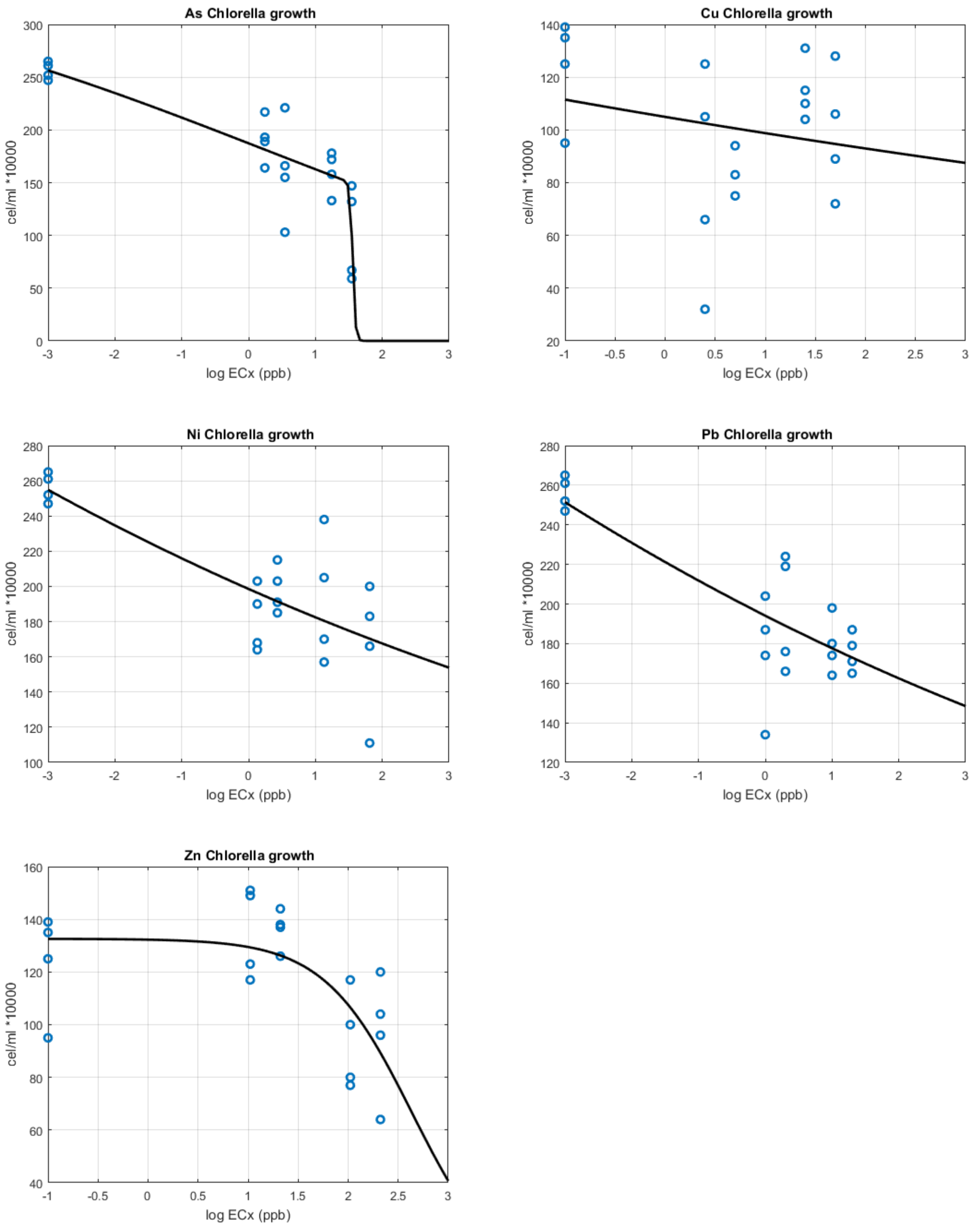

Figure S1. Concentration-response curves (lines) and experimental data (o) for C. vulgaris based on the growth inhibition results of single dilutions of heavy metals, taking into account the chemical species estimated by Eh-pH diagram (100% bioavailability). From top to bottom, curves of As, Cu, Ni, Pb and Zn. Hormetic model y = ((a- b)./(1+(exp((c* x)-

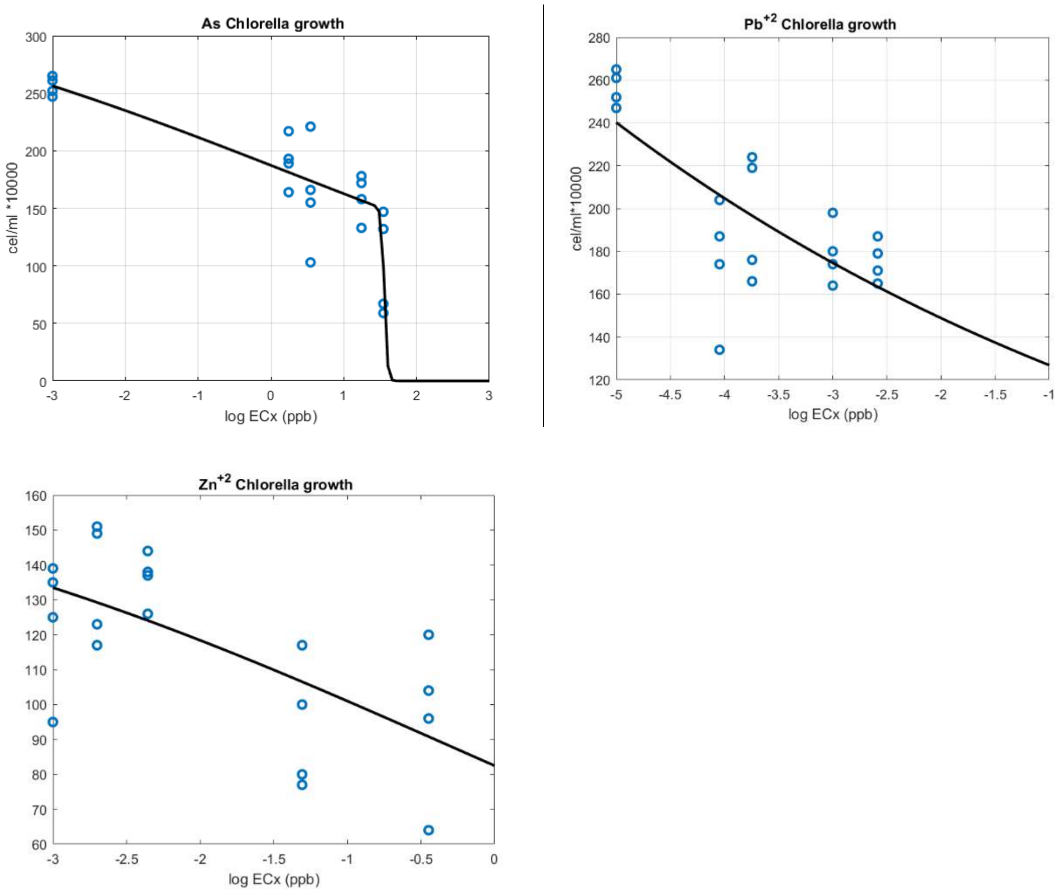

Figure S2. Concentration-response curves (lines) and experimental data (o) for C. vulgaris based on the growth inhibition results of single dilutions of heavy metals, taking into account the chemical species estimated by Visual MINTEQ. From top to bottom, curves of As (all species bioavailable), Pb and Zn. Hormetic model y = ((a- b)./(1+(exp((c* x)-d)))))./(1+(exp((e*x)- f))). DFE: 14. Fit parameters in Matlab: MaxFunEvals = 6000; MaxIter = 40000; Robust = 'Bisquare'.

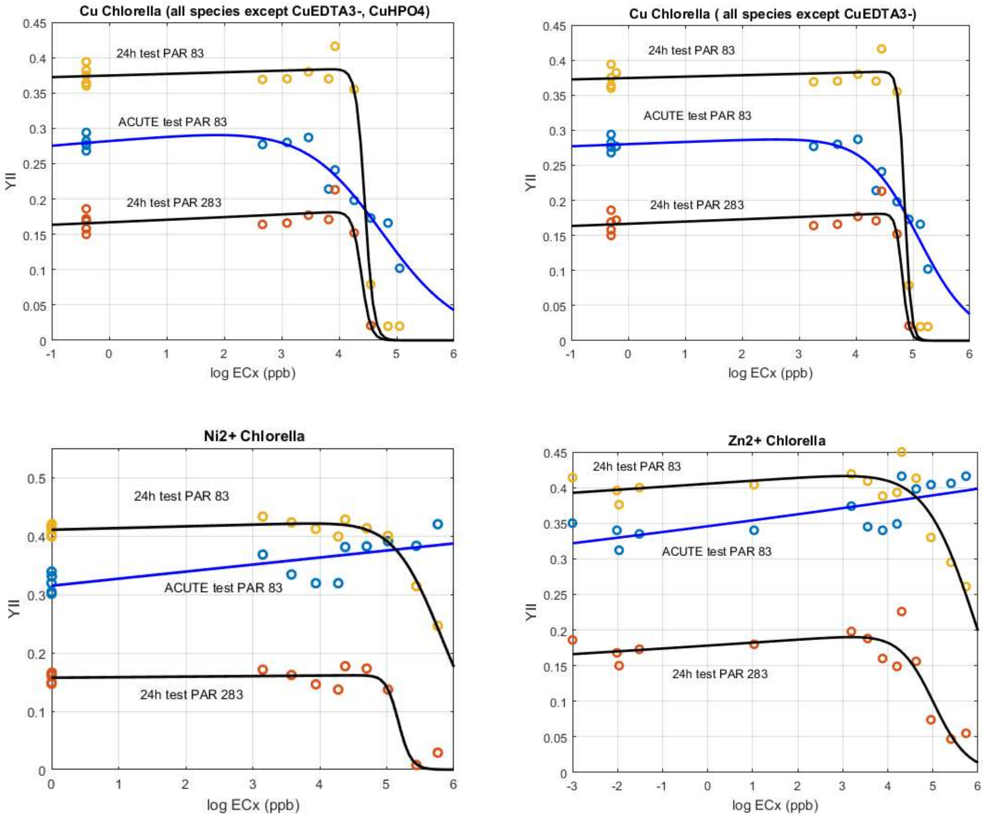

Figure S3. Concentration-response curves (lines) and experimental data (o) of YII for C. vulgaris based on the photosynthesis inhibition results of single dilutions of heavy metals, taking into account the chemical species estimated by Visual MINTEQ. From top to bottom, curves of Cu, Ni and Zn. Hormetic model y = ((a-b)./(1+(exp((c* x)-d)))).(1+(exp((e*x)- f))). DFE:8. Fit parameters in Matlab: MaxFunEvals = 6000; MaxIter = 40000; Robust = 'Bisquare'.

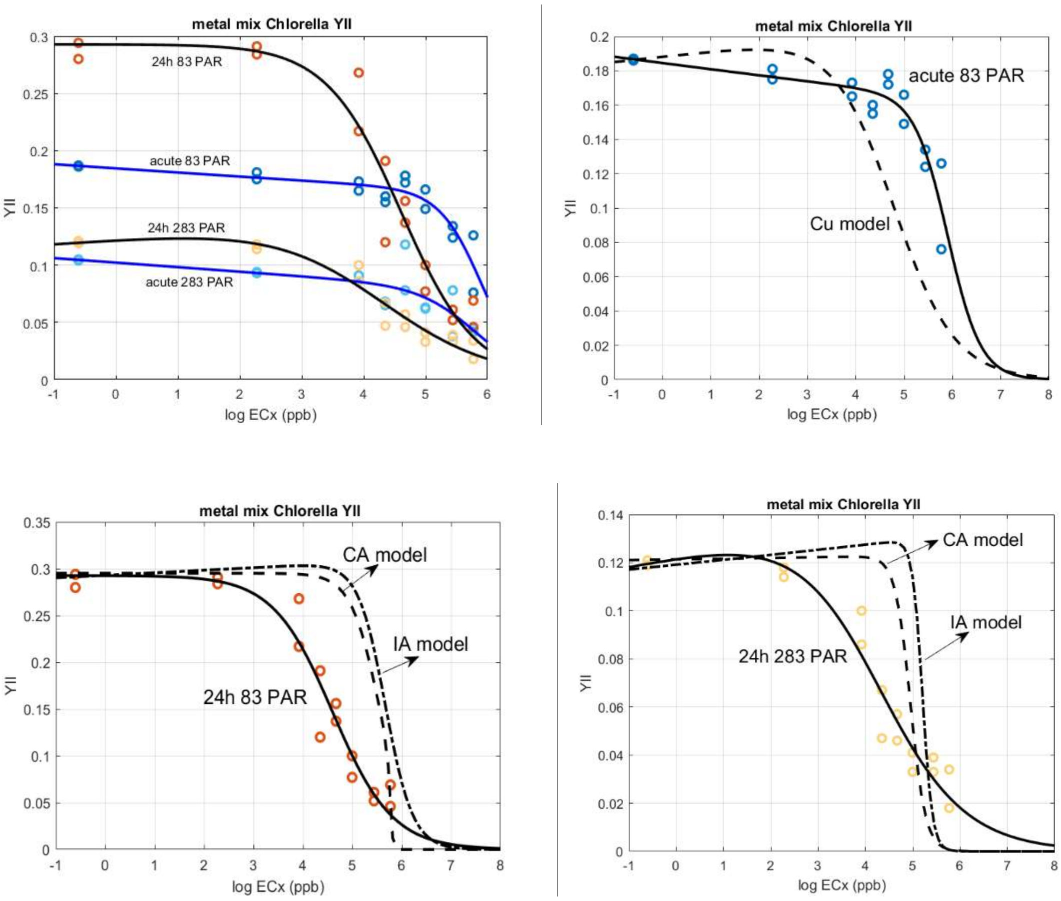

Figure S4. Concentration-response curves (lines) and experimental data (o) of YII for C. vulgaris based on the photosynthesis inhibition results of mixture dilutions of heavy metals, taking into account the chemical species estimated by Visual MINTEQ. Graphs with dashed curves indicates respective calculated Concentration Addition and Independent Action Model results. For acute effects only Cu showed measurable effects, so the experimental data are compared with Cu curve.
